# Phytoestrogens: Dietary Intake, Bioavailability, and Protective Mechanisms against Colorectal Neoproliferative Lesions

**DOI:** 10.3390/nu11081709

**Published:** 2019-07-24

**Authors:** Maria Teresa Viggiani, Lorenzo Polimeno, Alfredo Di Leo, Michele Barone

**Affiliations:** Gastroenterology Unit, Department of Emergency and Organ Transplantation (D.E.T.O.), University of Bari, Policlinic University Hospital, Piazza G. Cesare 11, 70124 Bari, Italy

**Keywords:** estrogen receptors, intestinal microbiota, familial adenomatous polyposis, colon adenomas, colorectal cancer

## Abstract

Phytoestrogens are natural substances that have been extensively studied for their beneficial effect on human health. Herein, we analyzed the data of the literature on the role of phytoestrogens in the prevention of colorectal neoproliferative lesions (CNL). Both in vitro and in vivo studies suggest that the beneficial effects of phytoestrogens on CNL mainly depend on their ability to bind estrogen receptor beta (ERβ) in the intestinal mucosa and counter ER-alpha (ERα) activity. Epidemiological data demonstrate a correlation between the low prevalence of CNL in Eastern populations and the consumption of soy products (phytoestrogen-enriched diet). However, both observational and interventional studies have produced inconclusive results. In our opinion, these discrepancies depend on an inadequate evaluation of phytoestrogen intake (dietary questionnaires were not aimed at establishing phytoestrogen intake) and absorption (depending mainly on the intestinal microbiota of the analyzed subjects). For this reason, in the present review, we performed an overview of phytoestrogen dietary intake and metabolism to offer the reader the opportunity for a better interpretation of the literature. Future prospective trials focusing on the protective effect of phytoestrogens against CNL should take into account both their dietary intake and absorption, considering the effective role of the intestinal microbiota.

## 1. Introduction

Phytoestrogens are natural substances that are able to exert estrogen-like activity thanks to their chemical structure similar to 17-β-estradiol. In particular, they have some typical beneficial properties of estrogens without producing their classical side effects (cerebral and cardiovascular accidents, higher incidence of endometrial and breast cancer). Global research over the last four decades has identified several benefits of food containing phytoestrogens. In fact, phytoestrogens have been used in the prevention of cardiac disease and menopausal symptoms, with an overall positive effect in subjects consuming moderate amounts of soy foods [1,2,3], while less conclusive results have been obtained in the treatment of osteoporosis [2]. For this reason, there has been an increase in soy products containing large amounts of dietary phytoestrogens in the human food market [1].

In addition to the previously mentioned health benefits of phytoestrogens, several studies support their protective effects against some hormone-sensitive cancer [4,5,6] and colorectal neoproliferative lesions [7]. This biological effect is mainly related to the ability of phytoestrogens to bind the estrogen receptor-beta, which is responsible for inhibitory activity on estrogen receptor-alpha related functions, including the stimulation of cell proliferation, and their ability to promote apoptosis [7].

In regard to colorectal neoproliferative lesions, while experimental studies strongly support the protective effect of phytoestrogens, clinical studies have produced conflicting results. This apparent discrepancy could be due to several reasons including the fact that these clinical studies have not been conceived to take adequate account of phytoestrogen intake (dietary questionnaires were not always aimed at establishing phytoestrogen intake) and absorption (phytoestrogen bioavailability is variable, depending mainly on the intestinal microbiota of the individuals). In order to better explain these aspects, in the present review, we will discuss the classification of phytoestrogens and their content in diet and biological mechanisms, provide a detailed description of their bioavailability, and finally examine their involvement in the different stages of colorectal neoproliferative lesions.

## 2. Phytoestrogen Classification

Phytoestrogens are classified into (1) isoflavones (genistein, daidzein, glycitein, biochanin A, and formononetin); (2) coumestans (coumestrol, wedelolactone, plicadin); (3) lignans (plant lignans: pinoresinol, lariciresinol, secoisolariciresinol, matairesinol, and enterolignans: enterodiol, enterolactone) [8,9,10,11]. They are also included in the larger structural class of polyphenols for the presence of phenolic groups in their structure [12]. Phytoestrogens mostly found in diet are isoflavones and lignans, and their main food sources are legumes, particularly soy, and in smaller quantities, they are contained in fruits, vegetables, and cereals [13,14].

## 3. Phytoestrogens and Diet

The data shown in Table 1 summarize the results obtained in five large epidemiological studies on the dietary intakes of isoflavones and lignans in various countries [15,16,17,18,19]. Subjects from European Mediterranean countries report the lowest average intake of phytoestrogens, whereas in Northern European countries the intake rises to 0.76 mg/day, with the exception of the United Kingdom where it reaches the amount of 4.04 mg/day [15]. However, in a more recent study conducted by Godos et al. [20] in a population from southern Italy, the mean intake of isoflavones and lignans was 4.0 ± 14 and 2.8 ± 2.6 mg/day, respectively. Nevertheless, Chinese and Japanese populations still have the highest isoflavone intakes worldwide due to their large consumption of soy products.

As regards the Mediterranean diet, it allows a low intake of isoflavones (predominantly through vegetables, legumes, sugar, coffee, and soy products) and a low intake of coumestrol (mainly through the consumption of coffee). On the other hand, lignans are assumed mainly through vegetables, fruit, and wine, and since these foods are well represented in the Mediterranean diet, lignans become the most abundant contributors of phytoestrogens [15].

Processed soy-based food products have been an integral part of regular diets in many countries of the Asia–Pacific region. However, in the last two decades, there have been concerted efforts to introduce soy products in Western diets, because of their potential health benefits [1]. On the contrary, Westernization of diet, with a dietary pattern shift towards increased intake of fat, sugar, and animal-source foods has been observed in Asian countries [21,22], probably causing a progressive reduction in traditional food intake.

## 4. Mechanism of Action of Phytoestrogens

Phytoestrogens may act by estrogen receptor (ER)-mediated genomic and non-genomic mechanisms. In general, phytoestrogens bind to estrogen receptors (ERs) and behave like weak agonists. However, though their affinity to ERs is 1/100 to 1/10,000 that of 17-β-estradiol, they may reach micromolar concentrations in the bloodstream, and this explains why they can act as both agonists and antagonists [23,24]. In addition, phytoestrogen biological activity can also be influenced by the level of endogenous estrogens and the type of ER receptor [25]. In fact, there are two distinct ERs named ER-alpha (ERα) and ER-beta (ERβ) (for a more comprehensive review see [7]). In vitro and in vivo studies conducted in ERβ knock out mice indicate that ERβ is a modulator of ERα activity since it is able to reverse the effects of ERα. The opposite biological role of the two receptors is described as the yin/yang relationship. In addition, it is known that ERα and ERβ have a different distribution in the various organs and systems, with ERβ being the prevalent form in the gut [7,26].

The isoflavone genistein has a 20- to 30-fold higher binding affinity for ERβ than for ERα, but the removal of one hydroxyl group from genistein results in daidzein, which has a much lower binding affinity for both ERs [27]. The conversion of the latter, indeed, leads to the formation of equol, which has a 10- to 100-fold greater estrogenic affinity than daidzein and a 10-fold greater estrogenic activity than genistein [24]. The greater binding affinity of phytoestrogens for ERβ compared to ERα explains why the administration of phytoestrogens does not produce the classic side effects associated with the administration of estrogens (cerebro/cardio-vascular events, increased risk of endometrial and breast cancer) [7]. For all these activities on ERs, phytoestrogens are defined as selective estrogen receptor modulators (SERMs) [28,29].

As ER-ligands, phytoestrogens can induce effects that do not depend on gene transcription or protein synthesis but are mediated by the modulation of membrane regulatory proteins, such as heterotrimeric G proteins and other transmembrane-spanning receptors, which involve second messengers and ultimately intracellular signals via epidermal growth factor (EGF) receptors [30,31]. Other non-genomic effects can involve mitogen-activated protein kinase, phosphatidylinositol trisphosphate kinase, and ion channel fluxes [32,33].

Using other mechanisms that are independent of their binding to ERs, phytoestrogens can also promote apoptosis and prevent the reproduction of malignant cells by blocking neoangiogenesis, tyrosine-kinase, and topoisomerase proteins. In addition, phytoestrogens have antioxidant activity, i.e., another biological activity that could indirectly exert an anticancer action [34,35]. However, these results were obtained, in most cases, using human cancer cell lines.

## 5. Intestinal Absorption and Metabolism of Phytoestrogens

**Figure 1:**
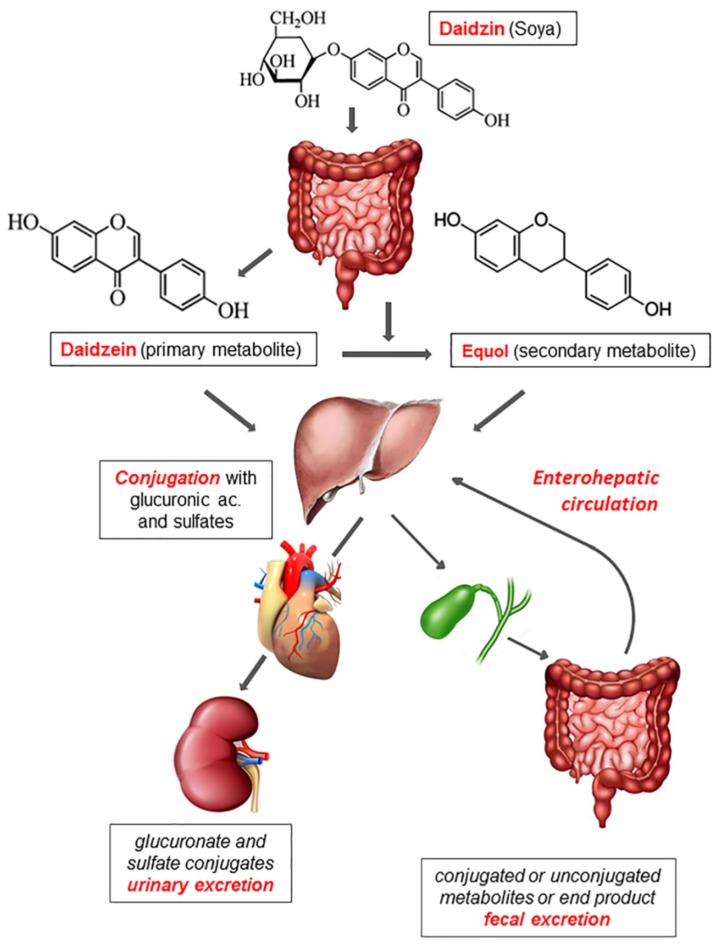
**Absorption, metabolism, and excretion of phytoestrogens**. Daidzin, contained in soy products, is hydrolyzed by the bacterial beta-glucosidase generating aglycones (primary metabolites). In addition, the colonic microflora is capable of transforming aglycones into secondary metabolites and bacterial end-products that are eliminated with feces. Both primary and secondary metabolites undergo either glucuronidation or sulfidation by intestinal epithelial cells and hepatocytes. Once in the bloodstream, these conjugated products reach target tissues and later on are excreted in urine or bile. In the latter case, they can be absorbed again by the intestine (enterohepatic circulation) or are excreted in feces as bacterial end-products or unconjugated forms.

To describe the intestinal absorption and metabolism of phytoestrogens, in Figure 1 we report, as an example, the absorption, metabolism, and excretion of the daidzin (the main component of soybeans), which belongs to the subgroup of phytoestrogens named isoflavones. Most phytoestrogens are present in diet as inactive glycoside conjugates (i.e., they are bound to glycidic molecules). Once ingested, these molecules are hydrolyzed only in a minimal part by the beta-glucosidase of the small intestine while bacterial beta-glucosidase of the colonic flora almost completes hydrolyzation, thus forming the so-called aglycones (primary metabolites) [8,9].

Thanks to the loss of their polar head, aglycones become liposoluble and are absorbed by the intestinal mucosa [10]. Lactobacilli, Bacteroides, and Bifidobacteria play an important role in the hydrolysis of isoflavones [36]. Moreover, the intestinal flora is capable of transforming aglycones into secondary metabolites that are more similar to estrogens and therefore have a higher affinity for ERs. A part of aglycones is catabolized by the intestinal flora generating end-products that are eliminated with feces [37,38].

After being absorbed by the intestinal mucosa, both primary and secondary aglycones are conjugated mainly with glucuronic acid and in part with sulfates through the action of UDP-glucuronyltransferase and sulfotransferases that operate at the level of the intestinal epithelium and the liver. These conjugated products are free to circulate in the bloodstream and exert their biological activity until they are excreted in urine and bile as conjugated glucuronides. When excreted in bile, they can be absorbed again by the intestine and enter enterohepatic circulation or are excreted in feces as unconjugated forms and bacterial end-products [10,39].

The urinary dosage of conjugated metabolites is used to determine the real absorption of phytoestrogens [10]. However, to find a detectable concentration of metabolites, the intake of a certain amount of phytoestrogens in the diet is needed, as observed in Eastern populations that traditionally eat soy and its derivatives. Moreover, the absorption, plasma concentration, and urinary excretion of phytoestrogens depend on the administered dose as well as on the relative bioavailability which, in turn, is influenced by sex (phytoestrogen metabolism is more efficient in women than in men), the intestinal transit time, and intestinal flora [40,41,42]. In fact, it has been found that the bioavailability of isoflavones varies from 13% to 35%, depending on the intestinal flora, and only 30%–40% of individuals, mostly Asians, and vegetarians, metabolize daidzein into equol [37,43,44], which is the phytoestrogen derivative with the highest affinity for ERs [41].

The importance of intestinal flora in the metabolism of phytoestrogens is based on the following observations: (1) the administration of antibiotics prevents the formation of active metabolites; (2) the presence of ileostomy determines low plasma and urinary levels of phytoestrogens; (3) infants fed with soy-enriched formulas during the first four months of life, when the intestinal flora is poorly developed, do not produce appreciable amounts of equol (active metabolite of daidzein) [10].

## 6. Equol

As already reported in Figure 1, equol is a daidzein metabolite derived essentially by the enzymatic activity of intestinal bacteria. In addition, to its high affinity for ERs, equol also has anti-androgenic properties and an antioxidant activity superior to all the phytoestrogens contained in the diet [45]. However, not all individuals who consume food containing daidzin produce equol. In fact, only 30–50% of the population is able to convert daidzein (the primary metabolite of daidzin) into equol, depending on intestinal flora composition [46]. Equol is only produced by anaerobic bacteria, among which are Eggerthella, Adlercreutzia, Asaccharobacter, Slackia, and Lactococcus [47].

Women with breast cancer presented lower, though not significant, urinary excretion of equol compared to healthy controls that consumed similar amounts of fibers within their diet [48]. The clinical efficacy of isoflavones, therefore, can depend on the ability to transform dietary isoflavones into equol.

## 7. Phytoestrogens and Sporadic Colon Adenomas

A study conducted by us on colon biopsies showed an ERβ reduction in adenomatous polyps as compared to the healthy mucosa [49]. This suggests that ERβ agonists may play a role in preventing adenomas and therefore colorectal cancer (CRC). In a Japanese case-control study (721 cases and 697 controls), lower dietary isoflavone intakes were associated with an increased risk of colon adenoma [50]. On the other hand, when an American population of patients with a history of colon adenomatous polyps (1905 patients) was studied, a reduction in the risk of advanced adenoma recurrence was demonstrated in those subjects with higher consumption of flavonols and not of all flavonoids [51]. Finally, our group carried out an interventional study on 60 patients with previous sporadic colon adenomas. In this study, we evaluated the effect of the administration of a compound (Eviendep^®^, Biohealth Italia srl, Rivoli, Italy) having as active ingredients two phytoestrogens (secoisolariciresinol and silymarin), on the ability to stimulate the expression of ERβ in colonic epithelial cells. Our findings confirmed our hypothesis since a significant increase of ERβ in colonic mucosal biopsies after two months of treatment was observed, suggesting a potential use of this phytoestrogen mixture in the prevention of adenoma recurrence [52].

## 8. Phytoestrogens and Familial Adenomatous Polyposis

Mice with a heterozygote mutation for the Apc gene (ApcMin/+) represent the most widely used animal model for experimental studies on familial adenomatous polyposis (FAP).

ApcMin/+ mice fed with a Western-type diet (at high risk for colorectal cancer onset due to the high-fat content and low fiber and calcium content) did not show a significant reduction in the number and size of polyps after administration of isoflavones [53]. However, in this study, all ApcMin/+ mice were obtained from mothers that were already kept on isoflavone-enriched diets from mating, throughout gestation and lactation (in-utero exposure), probably reducing the effect of the dietary treatment started at four weeks of age. Similarly, ovariectomized ApcMin/+ mice fed with a standard mouse diet did not show a significant reduction in the number and size of polyps after administration of genistein [54]. In this case, the lack of a beneficial effect was due in part to the use of genistein as the only bioactive compound, and in part to the use of a standard diet, which is not able to promote intestinal neoproliferative lesions as occurs after the administration of a high-fat/low-fiber diet. However, in this animal model, coumestrol showed a reduction in the number of polyps [54]. The mechanism by which coumestrol could reduce the formation of colon polyps is believed to be related to its higher affinity for binding to ERβ compared to ERα [27]. In fact, of the two known ERs, ERα is thought to have an estrogen-mediated proliferative activity, whereas ERβ is thought to be the antiproliferative form. ERβ is the predominant form in the gut, supporting ERβ as the most probable mediator of estrogenic antiproliferative effects in the colon [7,55]. ERβ deficiency enhances small intestinal tumorigenesis in ApcMin/+ mice [56]. As proof of concept, the administration of estrogens or the β-selective agonist coumestrol similarly blocked increased intestinal neoplasm development in ovariectomized ApcMin/+ mice [54]. Also, the phytoestrogens silymarin and lignin have also been shown to reduce polyp formation and determine an up-regulation of ERβ in ApcMin/+ mice treated with a high-fat/low-fiber content diet [57]. The peculiarity of this study consists in the use of male ApcMin/+ mice that did not undergo any surgical or hormonal manipulation, suggesting the possibility of a protective effect also in men. Ovariectomized ApcMin/+ mice have a lower ERα expression and a higher ERβ expression with 77% more intestinal tumors than non-ovariectomized ApcMin/+ mice. When 17-β-estradiol was administered, the number of intestinal tumors of the ovariectomized ApcMin/+ mice became similar to that of non-ovariectomized ApcMin/+ mice and the expression of ERα was further reduced [54]. Phytoestrogens with a higher affinity for ERβ, therefore, could decrease the formation of intestinal tumors when endogenous estrogens are deficient [58].

The importance of ERβ in the development of intestinal neoproliferative lesions in the ApcMin/+ model is confirmed by the antiproliferative effect of omega-3 and olive oil. In fact, their administration determined a reduction of intestinal polyp number and size that was associated with the significant increase of the ERβ/ERα ratio [59]. A further finding supporting the role of estrogen receptors in neoproliferative processes of colonic mucosa comes from our studies in patients with FAP [60]. FAP represents a model that summarizes the natural history of CRC since the simultaneous presence of “normal” mucosa, dysplastic lesions, and cancer can be detected in the same subject, excluding inter-individual biological variability [60]. In this study, there was a progressive and significant reduction of the ERβ/ERα ratio from normal mucosa to low-grade dysplasia, high-grade dysplasia, and finally, CRC [60], a finding observed also in humans with duodenal lesions in the course of FAP [61]. In another study carried out in humans with FAP, three month administration of Eviendep^®^ resulted in a reduction in the number (32%) and size (51%) of duodenal polyps in 11 patients that had undergone colectomy and ileal pouch construction [62].

## 9. Phytoestrogens and Colorectal Cancer (CRC)

Colorectal cancer (CRC) is the second most common cause of cancer death in Europe [63] and is more common in Western as compared to Eastern countries [13]. Epidemiological studies have correlated the low prevalence of CRC in Eastern populations with a diet rich in soy [13]. However, a more recent meta-analysis has reported that a dietary intake of isoflavones does not decrease the risk of colorectal cancer when prospective studies are considered, while it is significantly associated to reduced risk in case-control studies. In addition, the same authors reported that case-control studies on dietary lignan intake showed a significant lower risk for colorectal cancer [64].

A rapid increase in CRC incidence is now observed in Eastern Europe, Asia, and South America, while the incidence rate is stable or even declining in USA, Australia, New Zealand, and several Western European countries [21]. This shift in CRC trends has been interpreted as a consequence of the introduction of soy products in Western diets and the increase in fat, sugar, and animal-source food intake in Eastern countries and South America [1,21,22].

The previous epidemiological considerations justify the various studies on the effect of soy components, including phytoestrogens, on CRC carcinogenesis. In vitro and in vivo studies on the antitumor effect of phytoestrogens have produced promising results, suggesting the intervention of different biological mechanisms. One of these mechanisms consists in their binding with ERβ. It has been shown that the interaction of isoflavones with the ERβ induces antiproliferative and pro-apoptotic effects in the small intestine and colon [65,66], reducing CRC rates by 70–80% in ovariectomized female mice [67]. A further demonstration of the primary role of ERβ in CRC has been found in studies on animals in which the silencing of this receptor abolished the antiproliferative effect of isoflavones [65]. In contrast, the silencing of ERα produced anti-proliferative effects [67]. The inhibition of cell proliferation and the induction of apoptosis of intestinal cells mediated by genistein also occurs through mechanisms other than interaction with ERβ. In fact, genistein inhibits epidermal growth factor (EGF) activity while it activates the FOXO3 tumor suppressor gene and inhibits the insulin-like growth factor-I receptor (IGF-I) [68,69]. Moreover, phytoestrogen metabolites increase the activity of vitamin D that has well known antineoplastic and pro-differentiating effects [70].

Using the azoxymethane (AOM)/dextran-sulfate-sodium (DSS) animal model, which is widely used to study inflammation-associated colorectal cancer, we recently demonstrated that a diet enriched with phytoestrogens (the flavonolignan silymarin) and anti-inflammatory compounds (boswellic acids) exerts significant chemo-prevention due to inflammation reduction, epithelial turnover/apoptosis increase, and ERβ stimulation [71].

Numerous studies in humans have demonstrated a reduction of ERβ expression in CRC, which also correlated with the stage of the tumor [54,72,73,74]. These findings further supported the possible role of ERβ modulators in CRC prevention.

Several studies have evaluated the relationship between dietary intake of phytoestrogens and CRC in humans. Most of these data are summarized in some systematic reviews and meta-analyses. Jin et al. [75] analyzed eight studies on the role of flavonoids in the prevention of CRC, including four studies on isoflavones. A similar study was later performed by Woo et al. [76], who analyzed 23 primary studies, 9 of which were focused on isoflavones. Both studies concluded that no reliable implications for practice could be determined. Two more recent studies (a systematic review and a meta-analysis) focusing on phytoestrogens alone, found an association between isoflavone or lignin dietary intake, especially in Asian populations, with a reduced risk of CRC risk [77,78]. However, due to either the limited number of studies or their heterogeneity, all authors have concluded that the dietary intake of phytoestrogens in the prevention of CRC cannot be considered as a recommendation for public health [75,76,77,78].

Several reasons could explain why these reviews did not lead to conclusive results: (1) the difficulties in evaluating the intake of the compounds under investigation (some studies, especially on Western populations, did not estimate phytoestrogen intake through dietary questionnaires specifically aimed at establishing this aspect); (2) the wide qualitative and quantitative variations of the dietary intake in the studies; (3) the study design including different primary studies (case-control, cohort studies, clinical trials); (4) the populations considered (Asian or Western); (5) the type of subjects enrolled (both genders, wide age ranges, pre- and post-menopausal women), and the different length of the follow-up in the studies. In addition, it should be considered that the evaluation of the actual absorption of these nutrients (using plasma, serum, or urinary levels of phytoestrogen metabolites) is an essential datum considering that phytoestrogen bioavailability is variable (isoflavone absorption ranges from 13% to 35%, depending on the intestinal microflora of the subjects analyzed) [37]. Finally, CRC incidence in a population with a similar dietary pattern is influenced by the intestinal microbiota, an aspect that was not considered in these studies, although, together with the individual genetic background, it plays a pivotal role in the difference between responders and non-responders to dietary manipulation.

Before drawing the final conclusion, it is worth reporting the results of a case-control study that evaluated plasma phytoestrogen levels in two distinct Asian populations. In this study, an inverse relationship between isoflavone plasma levels and CRC risk, regardless of sex and anatomical subtype was found (*P* for trend = 0.043 and 0.001 for Korean and Vietnamese populations, respectively) [79].

## 10. Conclusions

Experimental in vitro and in vivo studies on the influence of phytoestrogens on CRC have been very promising. However, these results have not been fully confirmed by clinical studies which have produced some conflicting results. In our opinion, these apparent discrepancies are due, at least in part, to inadequate evaluation of phytoestrogen intake and absorption, since the latter depends on the intestinal microflora of the analyzed subjects. Future studies aimed to better clarify the potential protective effect of phytoestrogens against colorectal neoproliferative lesions should take into account both the intake and the absorption of these natural compounds. 

## Figures and Tables

**Table 1:** Comparison of dietary isoflavone and lignan intakes in various countries.

Countries	Isoflavones	Lignans
(mg/day per person, expressed as mean ± SD)		
**Mediterranean countries** [15] (Greece, Spain, Italy, and Southern France)	0.46 ± 0.05	1.02 ± 0.01
**Non-Mediterranean European countries** [15] (Northern France, Germany, the Netherlands, Denmark, Sweden, Norway)	0.76 ± 0.03	1.26 ± 0.01
**United Kingdom** [15]	2.34 ± 0.16	1.60 ± 0.04
**China** [16,17]	40.8 ± 28.7 (in women) 36.2 ± 24.4 (in men)	n.d.
**Japan (range)** [18,19]	20.8–46.2	n.d.

n.d.: not determined.
